# Robustness of the far-field response of nonlocal plasmonic ensembles

**DOI:** 10.1038/srep28441

**Published:** 2016-06-22

**Authors:** Christos Tserkezis, Johan R. Maack, Zhaowei Liu, Martijn Wubs, N. Asger Mortensen

**Affiliations:** 1 Technical University of Denmark, Department of Photonics Engineering, Kgs. Lyngby, 2800, Denmark; 2 University of California, San Diego, Department of Electrical and Computer Engineering, La Jolla, CA 92093-0407, USA; 3 Technical University of Denmark, Center for Nanostructured Graphene, Kgs. Lyngby, 2800, Denmark

## Abstract

Contrary to classical predictions, the optical response of few-nm plasmonic particles depends on particle size due to effects such as nonlocality and electron spill-out. Ensembles of such nanoparticles are therefore expected to exhibit a nonclassical inhomogeneous spectral broadening due to size distribution. For a normal distribution of free-electron nanoparticles, and within the simple nonlocal hydrodynamic Drude model, both the nonlocal blueshift and the plasmon linewidth are shown to be considerably affected by ensemble averaging. Size-variance effects tend however to conceal nonlocality to a lesser extent when the homogeneous size-dependent broadening of individual nanoparticles is taken into account, either through a local size-dependent damping model or through the Generalized Nonlocal Optical Response theory. The role of ensemble averaging is further explored in realistic distributions of isolated or weakly-interacting noble-metal nanoparticles, as encountered in experiments, while an analytical expression to evaluate the importance of inhomogeneous broadening through measurable quantities is developed. Our findings are independent of the specific nonclassical theory used, thus providing important insight into a large range of experiments on nanoscale and quantum plasmonics.

Plasmonics lies among the most prominent research fields in modern nanotechnology^1^,^2^,^3^, promising exciting applications and unravelling new phenomena as the length scale reduces^4^,^5^,^6^. Traditionally, noble metals constitute the material basis for novel plasmonic devices operating in the visible^7^, although many recent efforts are devoted to extensions towards the ultraviolet, infrared and THz parts of the spectrum^8^. A key issue in noble-metal plasmonics is its association with pronounced homogeneous broadening due to Ohmic losses in the metal^9^ and enhanced Landau damping near the surface^10^,^11^. Within classical electrodynamics, and in the quasistatic regime, radiation losses are small and the limited quality factor of plasmon resonances reflects material losses^12^. In other words, homogeneous broadening is important. Furthermore, the commonly employed local-response approximation (LRA) of classical electrodynamics predicts size-independent resonances for the nowadays experimentally accessible small nanoparticles (NPs) in the quasistatic regime^13^. As a consequence, despite the increasing impact of plasmonics and the promotion of single-particle spectroscopy^14^, little, if any, emphasis has been placed on the role of inhomogeneous broadening due to size distribution — even in experiments on NP ensembles with a noticeable size variation.

The observation of size-dependent resonance shifts not anticipated from classical electrodynamics has recently renewed interest in plasmons in the sub-10-nm regime^15^,^16^,^17^. State-of-the-art experiments range from single-particle spectroscopy with the aid of tightly focused electron beams^15^,^16^,^17^,^18^, to optical far-field measurements sampling the response of NP ensembles^19^,^20^,^21^,^22^,^23^. In the latter case, nonlocal effects^17^,^24^ and the concomitant inhomogeneous broadening can prove important for the interpretation of ensemble measurements. Ensemble averaging effects have been theoretically explored for exciton systems^25^, and for large-NP plasmonic collections dominated by retardation-driven redshifts^26^, but related studies in nonlocal plasmonics are still missing. The unambiguous observation of size-dependent resonance shifts in single-particle spectroscopy^15^,^17^,^27^ encourages therefore to explore broadening phenomena related to size distribution: *What is the robustness of plasmonic nonlocal effects when subject to ensemble averaging?*

The influence of ensemble spectral averaging on the far-field response of nonlocal plasmonic NP collections is studied here theoretically, starting with the ideal case of a normal distribution of free-electron, Drude-like nanospheres. Complexity is subsequently increased by considering more realistic distributions, resembling experimental histograms^28^, of noble-metal NPs, for which additional loss mechanisms like interband transitions and electron quantum confinement are important (the latter affects Drude NPs as well). Through detailed simulations within the framework of Mie theory and its appropriate extensions^13^,^24^,^29^,^30^, we show that ensemble averaging can have significant implications in more ideal cases, but becomes practically negligible when all mechanisms related to homogeneous broadening are taken into account in noble-metal plasmonics, a behaviour preserved even when weak interparticle interactions are taken into account. Our findings are therefore expected to provide additional flexibility to the design and analysis of experiments on the nanoscale: On the one hand, analysing the far-field response of a NP collection on the basis of the ensemble mean size is proven sufficient for the purposes of most experimental studies. On the other hand, nonlocal effects are not concealed by single-NP losses in large ensembles, thus allowing to connect with single-particle electron-energy-loss studies^15^,^17^.

## Results and Discussion

### Nonlocality-induced plasmon blueshifts

We first revisit the optical response of a small metallic nanosphere, embedded in air for simplicity. Our study is based on Mie theory^13^ and its appropriate extension for nonlocal effects (see the Methods section for more details)^31^,^32^,^33^. The metal is described as a free-electron plasma with transverse (*ε*_t_) and longitudinal (*ε*_1_) dielectric function components given by the frequency- (*ω*) and wave vector- (**k**) dependent Drude^13^ and hydrodynamic^34^,^35^ models, respectively


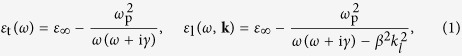


where *ω*_p_ is the plasma frequency of the metal, *ε*_∞_ is the background contribution of bound electrons and ions, *γ* is the damping rate, *β* the hydrodynamic parameter, and *k*_*l*_ the longitudinal wave number^35^. We take *ε*_∞_ = 1 and *γ* = 0.01 *ω*_p_ to focus on the role of free electrons and ensure low loss (associated with homogeneous broadening). We further assume 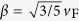 as obtained within the Thomas–Fermi theory^35^, where *v*_F_ (taken equal to 1.39 × 10^6^ m s^−1^ in the rest of the paper) is the Fermi velocity of the metal.

**Figure 1:**
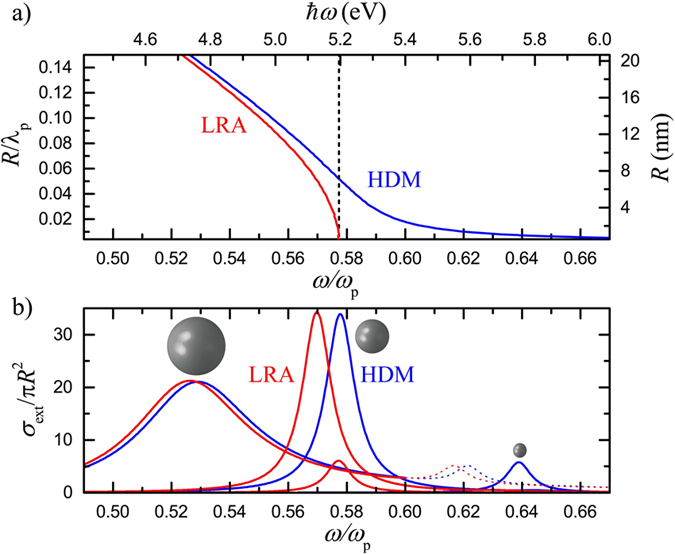
(**a**) Normalised frequency (*ω*/*ω*_p_) position of the dipolar plasmonic peak of a spherical NP described by the Drude model of equation (1), in air, as a function of its normalised radius *R*/*λ*_p_, obtained within the LRA (red line) and HDM (blue line) models. The black dashed line displays the prediction of the quasistatic approximation, 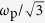. The corresponding energy in eV and radius in nm are given at the top and right axis respectively, assuming a plasmon energy *ħω*_p_ = 9 eV. (**b**) Extinction cross section (*σ*_ext_) spectra (normalised to the geometrical cross section *πR*^2^) for the NP of (**a**), for three radii, *R*/*λ*_p_ = 0.145, *R*/*λ*_p_ = 0.051, and *R*/*λ*_p_ = 0.007 (from left to right) within the LRA (red lines) and HDM (blue lines) models. For *ħω*_p_ = 9 eV these radii correspond to 20, 7, and 1 nm, respectively. The quadrupolar mode of the largest NP is depicted by thin dotted lines.

The size dependence of the frequency of the first (dipolar) plasmonic mode sustained by such a metallic nanosphere of radius *R* is plotted in Fig. 1a as obtained within LRA (*ω*_LRA_, red line) and the hydrodynamic Drude model (HDM) (*ω*_HDM_, blue line). To make our results scalable for different materials, *ω* and *R* are normalised to the plasma frequency and wavelength, *ω*_p_ and *λ*_p_ = 2*πc*/*ω*_p_ respectively. For a better illustration of the sizes and energies usually encountered, the corresponding plasmon energy (NP radius) is provided at the top (right) axis, assuming a typical value *ħω*_p_ = 9 eV^35^. For very small NP sizes, LRA reproduces the quasistatic result, 
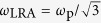
 (vertical dashed line in Fig. 1a). For larger sizes, retardation causes the modes to drastically redshift and become wider, as also observed in the normalised extinction cross section (*σ*_ext_) spectra of Fig. 1b (red lines corresponding to different NP sizes within LRA). Higher-order modes will not concern us here, and the quadrupolar plasmon peak of the largest sphere in Fig. 1b is only shown by thin dotted lines. The small-size modal frequency saturation predicted by LRA gives place to a continuous blueshift when the metal nonlocal response is taken into account. Comparison between LRA and HDM (blue lines in Fig. 1b) immediately shows that the frequency shifts become larger as the NP size decreases, but no additional resonance broadening due to nonlocality is observed.

A significantly different behaviour is expected in a statistical ensemble of small particles, where the strongly blueshifting modes of single NPs will overlap in a sequential manner, leading to important line broadening possibly even for narrow size distributions, in analogy to the effect of retardation on large NPs^26^. At this point we should also note that for the type of Drude metal described here, more detailed theories based on atomistic *ab initio* calculations predict frequency redshifts, instead of blueshifts, of similar magnitude, due to electron spill-out^10^,^36^,^37^,^38^. Indeed redshifts are measured for simple metals such as sodium^38^. Yet in noble metals such as silver and gold, the spill-out is less extended and the measured size-dependent blueshifts are well reproduced by HDM. An exact description of a specific material is beyond the scope of this paper, and simple nonlocal models should suffice for the study of ensemble averaging, regardless of the direction and origin of modal shifts.

### Inhomogeneous broadening in Drude-metal ensembles

**Figure 2:**
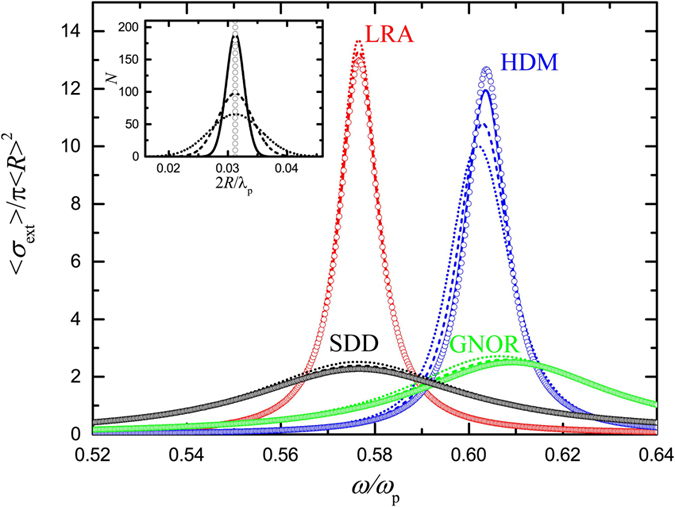
Averaged normalised extinction (〈*σ*_ext_〉) spectra calculated for *N* = 1000 NPs described by the dielectric function of equation (1) within the LRA (red lines), HDM (blue lines), GNOR (green lines) and SDD (black lines) models, for the size distributions shown in the inset. The average NP diameter is 2〈*R*〉/*λ*_p_ = 0.031, which for *ħω*_p_ = 9 eV corresponds to 4.3 nm, and the standard deviation of the normal distribution function is 0.2 (solid lines), 0.4 (dashed lines), and 0.6 (dotted lines). Open circles denote the corresponding spectra for the single mean-size NP, corresponding to the *δ*-function distribution (open circles) of the inset.

Ensemble spectral averaging is at a first step investigated by considering a collection of *N* = 1000 of the NPs described above, with a mean diameter 2〈*R*〉/*λ*_p_ = 0.031 (corresponding to 4.3 nm for *ħω*_p_ = 9 eV). The NP size follows normal distributions around this mean value as shown in the inset of Fig. 2, with standard deviations ranging from 0.2 (narrowest distribution, solid line) to 0.4 (dashed line) and 0.6 (widest distribution, dotted line). The extreme case of a *δ*-function distribution, i.e., all NP diameters corresponding precisely to the mean value, is depicted by open dots. This kind of *δ*-function distribution is exactly what one assumes in practice when disregarding ensemble averaging. We also note that, while the distributions of Fig. 2 are continuous functions, discrete size steps are taken in the simulations, small enough to achieve convergence of the averaged spectra. Apart from the LRA and HDM models, we also discuss calculations based on the commonly employed local size-dependent damping (SDD) model^39^ and the Generalized Nonlocal Optical Response (GNOR) theory^24^. Within SDD, the damping parameter *γ* becomes size dependent, *γ* → *γ* + *Av*_F_/*R*, to effectively take into account the experimentally observed single-NP damping^30^. The constant *A*, usually taken equal to 1 (as we do here) although a large range of values can be found in literature, is introduced to phenomenologically describe the reduction of the free-electron path length and to account to some extent for quantum-size corrections in very small NPs^19^,^39^,^40^,^41^,^42^. On the other hand, GNOR reproduces size-dependent damping in a more physical way, by incorporating electron diffusion as a measure of a variety of electron-scattering effects, including Landau damping due to generation of electron-hole pairs^43^. In practice, one needs merely to replace *β*^2^ in equation (1) with *β*^2^ + *D*(*γ* − i*ω*), where *D* is the diffusion constant of the metal. A thorough discussion on the determination of *D* can be found in a recent review by Raza *et al.*^35^; in general, it has to be chosen so as to reproduce the experimentally observed, and successfully reproduced by SDD models, plasmon damping. For the Drude-like NPs studied in this section, we find that the simple relation 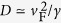
24 provides an excellent correspondence between the two models. However, it has been shown that more strict calculations are required in the case of noble metals^44^. While, in this respect, GNOR remains a phenomenological model, its strength is that, for arbitrarily shaped plasmonic NPs, it reproduces both the size-dependent blueshifts and the damping of plasmon modes by a simple correction in the dynamics of the free-electron fluid of HDM, whereas SDD models only capture the damping effects.

With these models at hand, we study in Fig. 2 how spectral averaging compares to single-NP response. Clearly, for the local models (LRA and SDD, red and black lines respectively), averaging does not practically affect the spectra. For all size distributions, the average extinction 〈*σ*_ext_〉, normalised to the geometrical cross section of the mean-size NP, *π*〈*R*〉^2^ (which is known in experiments), reproduces almost perfectly the spectrum of the individual mean-size NP, without frequency shifts or line broadening. Comparison with Fig. 1 shows that, in the size range of interest, local theories have already reached the quasistatic limit and the plasmon frequencies do not shift further, thus explaining the behaviour of the calculated spectra. The case becomes much different however when the spectra are size-dependent because of nonlocality, as is particularly pronounced by the HDM results. The incomplete spectral overlap for NPs of different sizes leads to an obvious broadening of the plasmon peaks, larger as the size distribution becomes wider. In addition, since larger NPs are characterised by larger extinction values, the overlap between large and small particles leads to a decrease of 〈*σ*_ext_〉, and to a gradual redshift of the ensemble resonance comparing with the single nonlocal mean-size NP. One may therefore conclude that statistical averaging can lead to significant deviations in experimental far-field measurements on ensembles of plasmonic NPs with wide size distributions. Nevertheless, since HDM disregards size-dependent damping mechanisms, it is crucial to take such effects into account. In view of the previous discussion, this is straightforward within GNOR (green spectra in Fig. 2). The differences between single-NP and ensemble response are now smoothed, leading to smaller additional modal shifts and almost negligible line broadening due to size inhomogeneity: the spectral width is mainly due to single-particle nonlocal broadening.

### Inhomogeneous broadening in noble-metal ensembles

**Figure 3:**
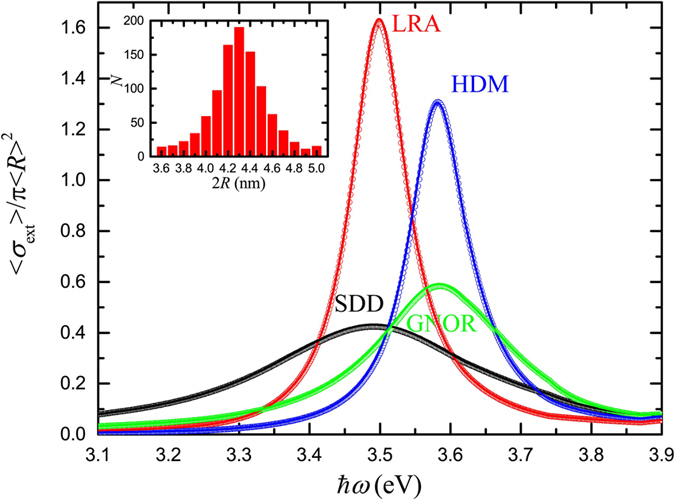
Averaged normalised extinction spectra calculated for *N* = 1000 silver NPs described by the experimental dielectric function of Johnson and Christy^45^ within the LRA (red line), HDM (blue line), GNOR (green line), and SDD (black line) models, for the size distribution shown by the histogram of the inset. The mean NP diameter is 2〈*R*〉 = 4.3 nm. Open circles denote the corresponding spectra for the single mean-size NP.

The important result of negligible effect of spectral averaging when single-NP size-dependent damping is taken into account may be appealing, but its validity was displayed only for ideal Drude metals and for normal size distributions. In order to connect with more practical, experimentally feasible situations, it is therefore important to carry out similar statistical studies for more realistic distributions in noble metals. We consider a collection of *N* = 1000 silver NPs, described by the experimental dielectric function (*ε*_exp_) of Johnson and Christy^45^, following the size distribution shown by the histogram of the inset of Fig. 3. In order to apply the HDM, SDD and GNOR models, we obtain *ε*_∞_ in equation (1) from the experimental values by subtracting the Drude part: 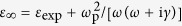, taking *ħω*_p_ = 8.99 eV and *ħγ* = 0.025 eV, values which describe bulk silver excellently. For SDD and GNOR we further assume *A* = 1 and 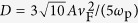, respectively^35^. The calculated spectra of Fig. 3 display now an almost negligible difference between single-NP and averaged spectra, even for the more pronounced in Fig. 2 HDM case. Homogeneous line broadening dominates the ensemble optical response, especially when single-NP size-dependent damping is taken into account within the more complete GNOR theory. This observation further strengthens our conclusion that inhomogeneous line broadening is not pronounced in most realistic NP ensembles (despite the non-negligible nonlocal response). Far-field optical experiments on small-NP ensembles can indeed be conducted for the observation of nonlocal frequency shifts, and their interpretation can be performed on the basis of the properties of the mean-size NP in the collection.

**Figure 4:**
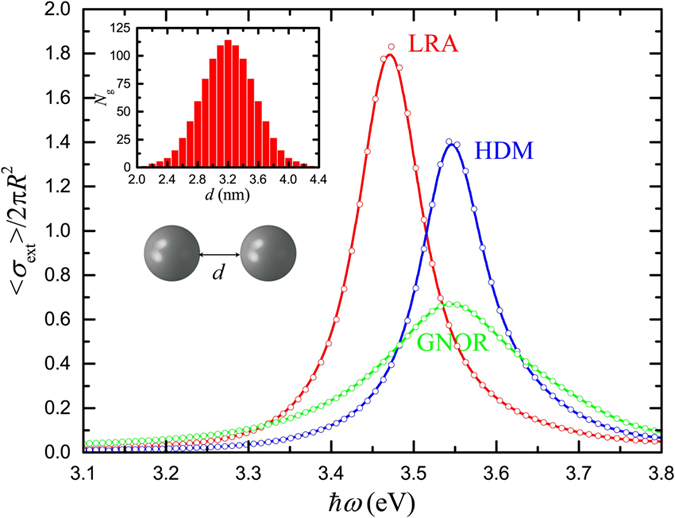
Averaged normalised extinction (〈*σ*_ext_〉/2*πR*^2^ here, since the geometrical cross section corresponds to the area occupied by two NPs) spectra calculated for *N*_g_ = 1000 silver NP dimers (both NPs have a diameter of 4.3 nm), separated by a gap of width *d*, as shown schematically in the inset, within the LRA (red line), HDM (blue line), and GNOR (green line) models. The gap width follows the normal distribution of the histogram of the inset, with a mean value 〈*d*〉 = 3.2 nm. Open circles denote the corresponding spectra for the single mean-gap dimer.

In addition to the study of isolated NPs, and the displayed robustness of their far-field optical response, an aspect that we have so far disregarded is the interaction between NPs in the ensemble. It is widely known that once plasmonic NPs are brought close to each other their interaction leads to significant modal redshifts, increasing as the interparticle gap is reduced^46^. A modified optical response is therefore expected for an ensemble of interacting small NPs, where two competing mechanisms, those of size-dependent blueshifts and interaction-induced redshifts are simultaneously present. The importance of this interplay is explored here, assuming that the average NP distance does not become smaller than *R*, thus preventing the particles from entering the nearly-touching regime, where purely quantum effects such as tunnelling become relevant^47^,^48^. Such distance control can be achieved nowadays with unprecedented precision, in dilute solutions with DNA- or ligand-functionalised NPs^20^,^49^,^50^,^51^. We assume *N*_g_ = 1000 dimers of identical, 4.3-nm silver NPs, separated by a gap of width *d*, as shown schematically in the inset of Fig. 4. The interparticle gap width follows a normal distribution around its mean value, 〈*d*〉 = 3.2 nm, ranging from 4.3 nm (a full NP width separating the two spheres) to 2.1 nm, as shown by the histogram of the inset. Comparison between Figs 3 and 4 shows that NP interaction can lead to a small plasmon redshift, of about 3–4 nm, both for the local case (only LRA is shown in Fig. 4 as the effect of SDD is well reproduced by its nonlocal counterpart, GNOR) and the nonlocal models. These relatively weak interactions owe their reduced strength to the small NP size and become important, according to our simulations, only for interparticle distances smaller than the NP radius. More importantly, the additional line broadening caused by such interactions is practically negligible, as it is immediately clear through comparison between Figs 3 and 4, and in any case it does not originate from the nonlocal optical response. It is therefore adequate, in most cases of practical interest, to disregard interactions and assume isolated NPs instead. Nevertheless, for a more strict description, it is still sufficient to take interactions into account through the average NP distance in the ensemble, as can be verified by the open dots in Fig. 4, which reproduce almost perfectly the gap-averaged spectra.

### Analytical evaluation of the importance of inhomogeneous broadening

Having considered situations where inhomogeneous broadening can be either strong or negligible, a simple way to decide on its importance without resorting to detailed simulations is desirable. To this end, we develop an analytical model which describes inhomogeneous broadening in terms of just the first two negative-order (or, with some further approximations, positive-order) moments of any NP-size distribution function. In practice, with a simple experimental size histogram at hand, one should be immediately able to tell whether the spectra are affected by inhomogeneous broadening. We begin by considering the dipole resonance in a single metallic NP, neglecting homogeneous broadening for the moment. Such a resonance can then be described by a spectral function 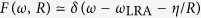, where *η* (∝*β* in our case) gives the strength of the leading-order 1/*R* correction associated with nonlocal response^33^. In an ensemble of non-interacting particles characterised by a size distribution *P*(*R*), the ensemble-averaged spectral function will be 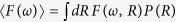. Our aim is to express the ensemble-averaged optical properties, such as the resonance frequency 〈*ω*〉, with the aid of the *n*th-order statistical moments of the particle ensemble, i.e. 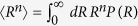. The homogeneous delta-function line shape allows to express the *n*th-order spectral moment 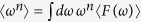 directly in terms of moments of the particle-size distribution,


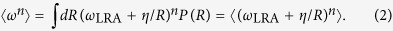


It is then straightforward to derive expressions for 〈*ω*〉 and the inhomogeneous broadening width, 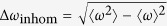, through the statistical moments of the particle-size distribution. As a key result, which allows to estimate the inhomogeneous broadening only in terms of the first two statistical moments of *P*(*R*) and the nonlocal blueshift *δω*_LRA→NL_ = 〈*ω*〉 − *ω*_LRA_ = *η*〈*R*^−1^〉 (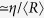 in a more crude approximation), it is shown that (see the related Discussion in the Supplementary Information)


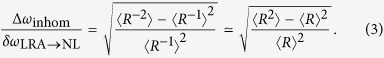


The first equality relates to the first and second negative-order moments of *P*(*R*), which are quite unusual ways of characterising a particle-size distribution – in most other contexts the positive-order moments (such as the mean value and variance) are the ones of interest. In the Discussion of the Supplementary Information we demonstrate the link between negative- and positive-order moments to obtain the second approximate identity in equation (3), which links directly to the relative particle-size fluctuation Δ*R*/〈*R*〉. This result holds for any description beyond classical electrodynamics that gives a 1/*R* leading-order blueshift of the LRA resonance frequency. Most importantly, it does not change if we replace *η* with −*η* to describe a corresponding 1/*R* redshift, so that our findings can be easily generalised to include other nonclassical effects, as anticipated above.

**Figure 5:**
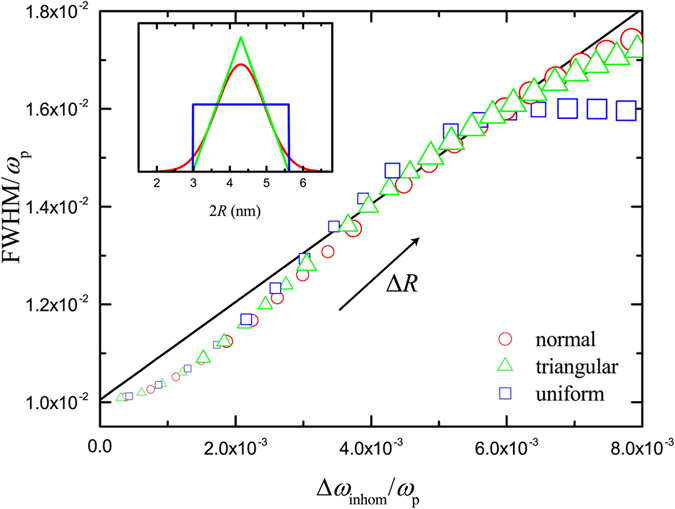
Parametric plot (open symbols) of Δ*ω*_inhom_ calculated from equation (3) versus FWHM obtained from simulations for a Drude-like NP within HDM (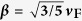, *γ* = 0.01*ω*_p_ in equation (1), for the size distributions shown in the inset. The average NP diameter is fixed at 2〈*R*〉/*λ*_p_ = 0.0312 (corresponding to 4.3 nm when *ħω*_p_ = 9 eV). Three different size distributions are plotted: uniform (blue line), triangular (green line) and (truncated) normal (red line). For the uniform (blue squares) and triangular (green triangles) cases, the distribution width increases from 0.13 · 10^−2^ to 2.80 · 10^−2^ (0.18 nm to 3.86 nm), while the standard deviation of the normal distribution (red circles) increases from 0.32 · 10^−3^ to 7.00 · 10^−3^ (0.044 nm to 0.965 nm). Increasing point size schematically depicts increasing distribution width. The black line denotes FWHM = Δ*ω*_inhom_ + FWHM_0_.

To test the validity of equation (3), we use it to evaluate Δ*ω*_inhom_ for certain distribution shapes and widths, assuming for simplicity *η* = *β*. The result is then compared to the full-width-half-maximum (FWHM) of the (averaged) plasmon peak calculated in each case by simulations performed for an ideal free-electron metal within HDM, with 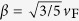 and *γ* = 0.01 *ω*_p_. As long as equation (3) holds, for different widths of the distribution, Δ*ω*_inhom_ is expected to follow a linear relation with FWHM. In Fig. 5 this is done for the three distributions shown in the inset: uniform, triangular and (truncated) normal. These examples are rather extreme situations, but in all cases an almost linear relation between Δ*ω*_inhom_ and FWHM, following the line FWHM = Δ*ω*_inhom_ + FWHM_0_ (black line in Fig. 5), where FWHM_0_ is the FWHM of the single mean-size NP, is indeed observed. For most distribution widths, all three examples give results that lie close to this line, indicating that the simple formula of equation (3) not only gives a good estimate of inhomogeneous broadening, regardless of the shape of the distribution, but can also be used to estimate the FWHM. It is worth noting that, for the uniform distribution, which is one of the most extreme situations to encounter in practice, larger deviations from the predictions of equation (3) are calculated as the distribution width becomes wider. This is due to the fact that, for wider distributions, a significant number of larger NPs is present in the ensemble. The extinction cross section of these NPs, which scales with *R*^3^, dominates the optical response, leading to a shifting of the averaged far-field response towards longer wavelengths. This effect is efficiently masked in more realistic situations, like the triangular and normal distributions of Fig. 5, for which larger NPs form just the tail of the distribution function, but cannot be neglected in a wide uniform distribution. Finally, it should also be stressed that, while the average NP size considered in Fig. 5 corresponds to 4.3 nm, the small-NP tails of the distribution functions are allowed to enter the sub-nm region, where classical or nonlocal electrodynamics are expected to fail, and approaches based either on quantum-corrected models^15^,^52^ or fully quantum-mechanical calculations^36^,^37^,^53^ should be employed. Nevertheless, such NP sizes, for which plasmonic effects are negligible and cluster fluorescence dominates instead^54^, concern only the tails of the widest distribution functions in Fig. 5, for which small deviations already start to appear. Consequently, calculating the corresponding spectra within HDM or GNOR will not practically affect our conclusions.

## Conclusion

In summary, the effect of inhomogeneous broadening of plasmon resonances due to nonlocal response in ensembles of small plasmonic NPs was explored through detailed simulations and analytical modelling. While inhomogeneous broadening is negligible in the LRA, it can be an important issue for Drude-like metals, especially within the standard HDM approach which neglects size-dependent damping in individual NPs. Crucially, however, ensemble averaging is shown to produce almost negligible deviations in most situations of practical interest, as illustrated for realistic size distributions of noble-metal NPs, and within the more accurate GNOR model. Nanoscale experiments involving large numbers of NPs can thus be designed and analysed in terms of the response of the mean-size NP in the ensemble, while far-field spectra of large NP collections are still expected to display the fingerprints of nonlocality, as in single-particle spectroscopies. We derived a simple equation to directly identify whether inhomogeneous broadening becomes important, simply through knowledge of the size distribution function in an ensemble. Our work provides therefore a valuable, general tool for the analysis of far-field optical spectra in modern experiments on plasmonics.

## Methods

### Nonlocal Mie theory

Here we summarise the fully-retarded Mie theory for a spherical plasmonic particle treated within HDM (and GNOR through a simple substitution of the hydrodynamic *β* parameter). The multipolar response of a sphere including nonlocal effects was determined by Ruppin^29^,^31^ by extending Mie theory^13^ to take into account the excitation of longitudinal waves. In the framework of Mie theory, the extinction cross section of a sphere of radius *R* embedded in a homogeneous host medium is given by^13^


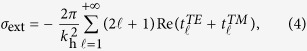


where 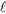 denotes the angular momentum and *k*_h_ is the wave number in the host medium, which is described by a dielectric function *ε*_h_. Assuming that the magnetic permeabilities, both in the sphere and in the host medium are equal to 1, the nonlocal Mie scattering coefficients are^29^,^31^,^32^,^33^


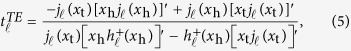



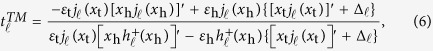


where 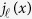 and 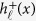 are the spherical Bessel and first-type Hankel functions, respectively, while *x*_h_ = *k*_h_*R* and *x*_t_ = *k*_t_*R*. Here *k*_t_ is the (transverse) wave number inside a sphere described by a transverse dielectric function *ε*_t_. The nonlocal correction 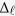 to the Mie coefficients is given as


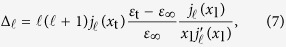


where *x*_1_ = *k*_1_*R* and *k*_1_ is the longitudinal wave number in the sphere, associated with the longitudinal dielectric function *ε*_1_, which is frequency- and wave vector-dependent. The dispersion of longitudinal waves is given by *ε*_1_(*ω*, **k**) = 0. In the limiting case where 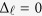 we retrieve the local result of standard Mie theory. All numerical results for isolated NPs have been obtained from numerical evaluations of equation (4). The corresponding results for NP dimers were obtained by use of a commercial finite-element method solver (COMSOL Multiphysics 5.0, RF module), using the appropriate extension to include nonlocal effects^55^,^56^.

## Additional Information

**How to cite this article**: Tserkezis, C. *et al.* Robustness of the far-field response of nonlocal plasmonic ensembles. *Sci. Rep.* **6**, 28441; doi: 10.1038/srep28441 (2016).

## Supplementary Material

Supplementary Information
