# Supplementary material for: Robustness of the far-field response of nonlocal plasmonic ensembles
Source: Sci Rep. 2016 Jun 22;6:28441. doi: 10.1038/srep28441 (PMC4916464; doi:10.1038/srep28441)
Supplement: Supplementary Information [file srep28441-s1.pdf]

Robustness of the far-field response of nonlocal plasmonic ensembles: Supplementary Information

Christos Tserkezis[1,*], Johan R. Maack[1], Zhaowei Liu[2], Martijn Wubs[1,3], and N. Asger Mortensen[1,3,*]

[1]Technical University of Denmark, Department of Photonics Engineering, Kgs. Lyngby, 2800, Denmark
[2]University of California, San Diego, Department of Electrical and Computer Engineering, La Jolla, CA 92093-0407, USA
[3]Technical University of Denmark, Center for Nanostructured Graphene, Kgs. Lyngby, 2800, Denmark
*ctse@fotonik.dtu.dk; asger@mailaps.org

Supplementary Discussion

Derivation of equation (3) of the main text

In the main text we have defined the inhomogeneous broadening width as

$$\Delta\omega_{\mathrm{inhom}} = \sqrt{\langle\omega^2\rangle - \langle\omega\rangle^2} \tag{S1}$$

From equation (2) of the main text, the first- and second-order moments of ω are

$$\langle\omega\rangle = \langle(\omega_{\mathrm{LRA}} + \eta/R)\rangle \tag{S2}$$

and

$$\langle\omega^2\rangle = \langle(\omega_{\mathrm{LRA}} + \eta/R)^2\rangle, \tag{S3}$$

respectively. Then

$$\Delta\omega_{\mathrm{inhom}} = \sqrt{\left\langle\left(\omega_{\mathrm{LRA}} + \frac{\eta}{R}\right)^2\right\rangle - \left\langle\left(\omega_{\mathrm{LRA}} + \frac{\eta}{R}\right)\right\rangle^2} = \sqrt{\left\langle\omega_{\mathrm{LRA}}^2 + \frac{2\,\omega_{\mathrm{LRA}}\eta}{R} + \frac{\eta^2}{R^2}\right\rangle} = \eta\sqrt{\left\langle\frac{1}{R^2}\right\rangle - \left\langle\frac{1}{R}\right\rangle^2}. \tag{S4}$$

In the above we have taken into account that (naturally) $\langle\omega_{\mathrm{LRA}}\rangle = \omega_{\mathrm{LRA}}$ and $\langle\eta\rangle = \eta$. Using $\delta\omega_{\mathrm{LRA}\to\mathrm{NL}} = \eta\langle R^{-1}\rangle$, it is then straightforward to derive equation (3) of the main text.

Statistical moments: relating negative to positive moments

For a narrow distribution function $P(R)$, without significant small- and large-particle tails, the negative-order moments appearing in the first equality of equation (3) of the main text can be expressed in terms of the more common positive-order moments, to give the approximate result on the right-hand side of equation (3) of the main text. This challenge is illustrated in Supplementary Fig. 1.

For the first negative-order moment, R^{-1} can be expressed as a Taylor series expanded around the average of the distribution, $R_0 = \langle R\rangle$,

$$\frac{1}{R} = \sum_{n=0}^{+\infty} \frac{1}{R_0^{n+1}} (R_0 - R)^n = \frac{1}{R_0} - \frac{1}{R_0^2}(R - R_0) + \frac{2}{2R_0^3}(R - R_0)^2 \ldots, \tag{S5}$$

and then the moment $\langle R^{-1} \rangle$ can be calculated with

$$\langle R^{-1} \rangle = \int_{-\infty}^{+\infty} R^{-1} P(R) dR = \int_{-\infty}^{+\infty} \sum_{n=0}^{+\infty} \frac{1}{R_0^{n+1}} (R_0 - R)^n P(R) dR. \tag{S6}$$

In this expression, R^{-1} and the Taylor expansion go to infinity when $R = 0$, which may cause the integral to diverge. It must be therefore required that $P(R) = 0$ for $R \leq 0$, which occurs of course for any realistic function $P(R)$. Furthermore, the summation is performed over infinite terms, and there is no immediate reason to truncate it. In fact, if $R > 2R_0$ each subsequent $n + 1$ term in the sum will be larger in absolute value than the previous, n, and of opposite sign. To be able to truncate this series, we must ensure that each $n + 1$ term is smaller than the previous one, and this is done by requiring that $P(R) = 0$ for $R \geq 2R_0$.

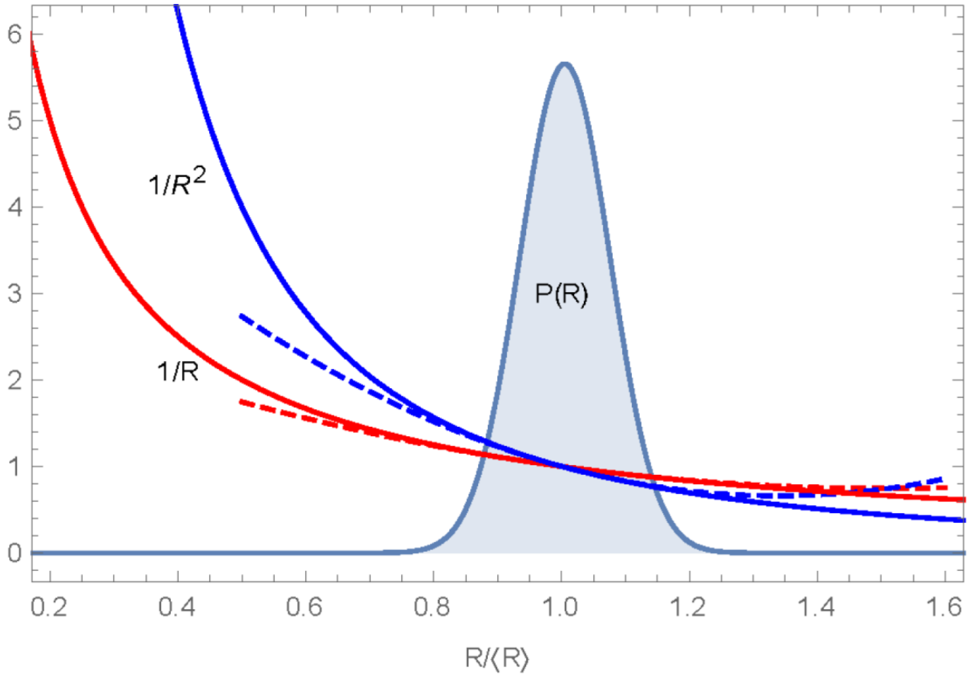

Supplementary Figure 1: Taylor series approximation of negative-order moments for a narrow distribution function. The dashed lines illustrate Taylor series approximations to the first and second negative-order moments, see equations (S7) and (S10).

We can now derive an approximate result for $\langle R^{-1} \rangle$. By including the first three terms of the series we get

$$\frac{1}{R} \simeq \frac{1}{R_0} - \frac{R-R_0}{R_0^2} + \frac{(R-R_0)^2}{R_0^3}, \tag{S7}$$

which implies that

$$\langle \frac{1}{R} \rangle \simeq \frac{1}{R_0} + \frac{\langle (R-R_0)^2 \rangle}{R_0^3}, \tag{S8}$$

and consequently (neglecting high-order terms)

$$\langle \frac{1}{R} \rangle^2 \simeq \frac{1}{R_0^2} + \frac{2\langle (R-R_0)^2 \rangle}{R_0^4}. \tag{S9}$$

Likewise, for the second negative-order moment we Taylor expand $1/R^2$ around R_0 to get

$$\frac{1}{R^2} \simeq \frac{1}{R_0^2} - \frac{2(R-R_0)}{R_0^3} + \frac{3(R-R_0)^2}{R_0^4}, \tag{S10}$$

which in turn implies that

$$\langle\frac{1}{R^2}\rangle \simeq \frac{1}{R_0^2} + \frac{3\langle(R-R_0)^2\rangle}{R_0^4}. \tag{S11}$$

Then, for the size fluctuations we have

$$\langle\frac{1}{R^2}\rangle - \langle\frac{1}{R}\rangle^2 \simeq \frac{\langle(R-R_0)^2\rangle}{R_0^4}, \tag{S12}$$

and thus

$$\sqrt{\langle\frac{1}{R^2}\rangle - \langle\frac{1}{R}\rangle^2} \simeq \frac{1}{R_0}\frac{\sqrt{\langle(R-R_0)^2\rangle}}{R_0} = \frac{1}{\langle R\rangle}\frac{\sqrt{\langle R^2\rangle - \langle R\rangle^2}}{\langle R\rangle}. \tag{S13}$$

Uniform distribution

As a particular example that can be treated analytically, we consider a uniform distribution function

$$P(R) = \frac{1}{\delta R}\theta(R - R_0 + \delta R/2)\theta(-R + R_0 + \delta R/2), \tag{S14}$$

where $\theta(x)$ is the Heaviside function. By construction, $P(R)$ is normalised and with a mean value of $\langle R\rangle = R_0$, while $\langle(R - R_0)^2\rangle = \frac{1}{12}(\delta R)^2$. The requirement that all radii in the distribution are positive gives a bound on its parameters, namely that $R_0 \geq \delta R/2$. For the first negative-order moment we get

$$\langle R^{-1}\rangle = \frac{1}{\delta R}\int_{R_0-\delta R/2}^{R_0+\delta R/2} dR\, R^{-1} = \langle R\rangle^{-1} g_1(\frac{\delta R}{\langle R\rangle}), \tag{S15}$$

with

$$g_1(x) = x^{-1}\ln\left(\frac{2+x}{2-x}\right) = 1 + \frac{1}{12}x^2 + \mathcal{O}(x^4). \tag{S16}$$

Similarly, for the second negative-order moment we get

$$\langle R^{-2}\rangle = \frac{1}{\delta R}\int_{R_0-\delta R/2}^{R_0+\delta R/2} dR\, R^{-2} = \langle R\rangle^{-2} g_2(\frac{\delta R}{\langle R\rangle}), \tag{S17}$$

with

$$g_2(x) = \frac{4}{4-x^2} = 1 + \frac{1}{4}x^2 + \mathcal{O}(x^4). \tag{S18}$$

In this way we can directly calculate

$$\sqrt{\langle\frac{1}{R^2}\rangle - \langle\frac{1}{R}\rangle^2} \simeq \frac{1}{\sqrt{12}}\frac{\delta R}{R_0^2}. \tag{S19}$$

Returning to Supplementary equation (S13) we indeed find the same result. In a similar way, for the case of the triangular and normal distribution that concern us in the main text, the result is

Triangular: $\sqrt{\langle\frac{1}{R^2}\rangle - \langle\frac{1}{R}\rangle^2} \simeq \frac{1}{\sqrt{24}}\frac{\delta R}{R_0^2}$ (S20)

Normal: $\sqrt{\langle\frac{1}{R^2}\rangle - \langle\frac{1}{R}\rangle^2} \simeq \frac{\sigma}{R_0^2}$, (S21)

where σ is the standard deviation of the normal distribution. Note that the normal distribution is truncated, limited in the region $R = 0 - 2R_0$.
